# Genome-Wide Identification and Characterization of the *CsFHY3/FAR1* Gene Family and Expression Analysis under Biotic and Abiotic Stresses in Tea Plants (*Camellia sinensis*)

**DOI:** 10.3390/plants10030570

**Published:** 2021-03-17

**Authors:** Zhengjun Liu, Chuanjing An, Yiqing Zhao, Yao Xiao, Lu Bao, Chunmei Gong, Yuefang Gao

**Affiliations:** 1 College of Horticulture, Northwest A&F University, Xianyang 712100, China; liuzj@nwafu.edu.cn (Z.L.); zyq2019@nwafu.edu.cn (Y.Z.); baolu@nwafu.edu.cn (L.B.); gcm228@nwafu.edu.cn (C.G.); 2 State Key Laboratory of Natural and Biomimetic Drugs, Department of Chemical Biology, School of Pharmaceutical Sciences, Peking University, Beijing 100191, China; anchuanjing@bjmu.edu.cn; 3 Department of Foreign Languages, Northwest A&F University, Xianyang 712100, China; 65356457@qq.com

**Keywords:** CsFHY3/FAR1 family, light, biotic and abiotic stress, expression pattern, interaction network

## Abstract

The *FHY3/FAR1* transcription factor family, derived from transposases, plays important roles in light signal transduction, and in the growth and development of plants. However, the homologous genes in tea plants have not been studied. In this study, 25 *CsFHY3/FAR1* genes were identified in the tea plant genome through a genome-wide study, and were classified into five subgroups based on their phylogenic relationships. Their potential regulatory roles in light signal transduction and photomorphogenesis, plant growth and development, and hormone responses were verified by the existence of the corresponding *cis*-acting elements. The transcriptome data showed that these genes could respond to salt stress and shading treatment. An expression analysis revealed that, in different tissues, especially in leaves, *CsFHY3/FAR1s* were strongly expressed, and most of these genes were positively expressed under salt stress (NaCl), and negatively expressed under low temperature (4 °C) stress. In addition, a potential interaction network demonstrated that PHYA, PHYC, PHYE, LHY, FHL, HY5, and other FRSs were directly or indirectly associated with CsFHY3/FAR1 members. These results will provide the foundation for functional studies of the CsFHY3/FAR1 family, and will contribute to the breeding of tea varieties with high light efficiency and strong stress resistance.

## 1. Introduction

As an indispensable environmental factor, light is involved in many biological processes, including plant growth and development, photomorphogenesis, chlorophyll biosynthesis, and chloroplast development [1,2]. In order to ensure their normal growth and development, higher plants have evolved sophisticated and multiple photoreceptors which can sense and adapt to the light environment, such as phytochromes, cryptochromes, phototropins, and ultraviolet-B (UV-B) receptors [3,4,5]. In Arabidopsis, there are five phytochromes (PHY) encoded by five specific genes, that is, *PHYA-PHYE*. Among the five phytochromes, PHYA is the primary photoreceptor which is responsible for perceiving and mediating various far-red light-mediated responses [6], whereas PHYB functions mainly in regulating the light responses to red light [7,8]. The active form of PHYA is translocated to the nucleus in order to perform its activity through interactions with small FAR-RED ELONGATED HYPOCOTYL1 (FHY1) or FHY1-like (FHL) proteins, which contain the nuclear targeting sequence [9]. The nuclear accumulation of PHYA will further promote downstream transcription activity and enhance the subsequent responses [10]. Upstream of FHY1/FHL, FAR-RED ELONGATED HYPOCOTYL3 (FHY3) and its homologous gene FAR-RED-IMPAIREDRESPONSE1 (FAR1) can form a homodimer or heterodimer, and can directly activate the transcription of *FHY1/FHL* under far-red light by binding to the promoters of *FHY1* and *FHL*. Consequently, FHY3/FAR1 are also considered to be important regulators in the PHYA signaling pathway [11]. In addition, ELONGATED HYPOCOTYL5 (HY5) was shown to interact with FHY3 and FAR1 by binding ACGT-containing elements in the *FHY1/FHL* promoters, and working together with CIRCADIAN CLOCK ASSSOCIATED1 (CCA1) and LATEELONGATED HYPOCOTYL (LHY) to generate the rhythmic expression of *EARLY FLOWERING4* (*ELF4*) [12,13]. Evolutionary studies have shown that the FHY3/FAR1 family is derived from transposases, which are specific transcription factors for plants [14,15].

In total, 14 FHY3/FAR1-related sequence (FRS) proteins have been identified in Arabidopsis [15]. A phylogenetic analysis showed that AtFHY3/FAR1 family members can be divided into five groups: group I (FHY3, FAR1, FRS1, FRS2 and FRS4), group II (FRS6 and FRS8), group III (FRS7 and FRS12), group IV (FRS3, FRS5 and FRS9), and group V (FRS10 and FRS11) [9,16]. Although the protein sequence of the MURA transposase encoded by maize *Mutator* was identified in AtFHY3/FAR1, terminal inverted repeats and other transposon-like structures were not found; therefore, the transposon activities of AtFHY3/FAR1 were likely to be lost [14,17]. Most of the AtFHY3/FAR1 family proteins contain three functional domains: the N-terminal C2H2 zinc finger with a DNA-binding domain (FAR1 DNA-binding domain), which can recognize specific DNA motifs in the transposon terminal reverse repeat sequence; the central putative core transposase domain (MULE domain); and the C-terminal SWIM motif, which has a transcriptional activation domain, while FRS9 has no FAR1 protein-binding domain, and FRS7 and FRS12 have two FAR1 protein-binding domains (SWIM domain) [15,18].

Among the AtFHY3/FAR1 family, FHY3 and FAR1 have been widely studied in Arabidopsis. ChIP-seq and DNA affinity purification sequencing (DAP-seq) analysis confirmed that FHY3 and FAR1 specifically bind to FBS (CACGCGC) *cis*-elements of more than 1000 gene promoter regions [19,20,21]. In addition, FRS4/CPD25 has also been shown to bind to FBS or FBL (FBS-like) *cis*-elements in the *ACCUMULATION AND REPLICATION OF CHLOROPLASTS5* (*ARC5*) promoter [22]. Although AtFRS9 does not have a FAR1-binding domain, it is assumed that it might interact with other FHY3/FAR1 members [15]. Through the expression regulation of the downstream target genes, AtFHY3/AtFAR1 was reported to play important roles in light signal transduction, and plant growth and development [11,16], e.g., photomorphogenesis [12], the circadian clock [23], branching and flowering time [24,25], chlorophyll biosynthesis [26], chloroplast division [21,22], starch synthesis [27], the abscisic acid response [28], the oxidative stress response [29], plant immunity [30], the low phosphorus response [31], and the growth and defense response balance [32,33]. Although many functions of FAR1 and FHY3 have been identified, it was found that FAR1 and FHY3 play more roles than expected, and the functions of other members of the *FHY3/FAR1* family are still not clear. Therefore, further studies on FRS proteins and other species of *FHY3/FAR1* family proteins still need to be carried out.

Tea (*Camellia sinensis* (L.) O. Kuntze) is an important economic crop which originates from China, and is the favorite non-alcoholic beverage of the world [34,35]. The growth environment of tea plants includes a warm temperature and humid and acid conditions. The tea plant is susceptible to various biotic and abiotic stresses, especially temperature and light, which affects its primary and secondary metabolites, yield, and quality [36]. The *FHY3/FAR1* family has been proven to play important roles in the growth and development of plants. So far, there has been no report on the *FHY3/FAR1* family in tea plants. It is valuable to identify the *FHY3/FAR1* family in tea plants, and to clarify its functions. In this study, 25 *CsFHY3/FAR1* family genes were identified in the tea plant genome via a genome-wide study, and were analyzed based on bioinformatics, including their basic characteristics, phylogenetic relationships, gene structure, conserved motifs, functional interaction network, and a transcriptome analysis. In addition, an expression analysis of the different tissues of the tea plants and *CsFHY3/FAR1* families under various biotic and abiotic stresses, including high temperature (40 °C), low temperature (4 °C), salt, drought, and abscisic acid (ABA), revealed the molecular characteristics of the *CsFHY3/FAR1* gene family, which provides a theoretical basis for further study of the biological function of CsFHY3/FAR1.

## 2. Results

### 2.1. Identification and Analysis of CsFHY3/FAR1 Genes

A total of 25 putative *CsFHY3/FAR1* genes were retrieved from the tea plant genome [37], named *CsFRS-1* to *CsFRS-25*. Their individual characteristics—including their coding DNA sequences (CDS), protein sequences, cellular location, and physiological and biochemical properties—are summarized in Table 1 and Table S2. As shown in Table 1, the molecular weight (MW) of the proteins ranged from 43.19 kDa (CsFRS-7) to 103.05 kDa (CsFRS-10), and the pI values ranged from 5.79 (CsFRS-3) to 9.28 (CsFRS-8). Furthermore, the subcellular location information indicated that most members were predicted to target the nucleus in order to perform their functions. It is noteworthy that several members, such as CsFRS-1, CsFRS-7, CsFRS-14, CsFRS-16, CsFRS-18, and CsFRS-23, were predicted to be located in the chloroplast and/or cytoplasm, which suggested the evolution of potentially new functions in these locations for these proteins.

### 2.2. Phylogenetic Analysis of CsFHY3/FAR1s

**Figure 1:**
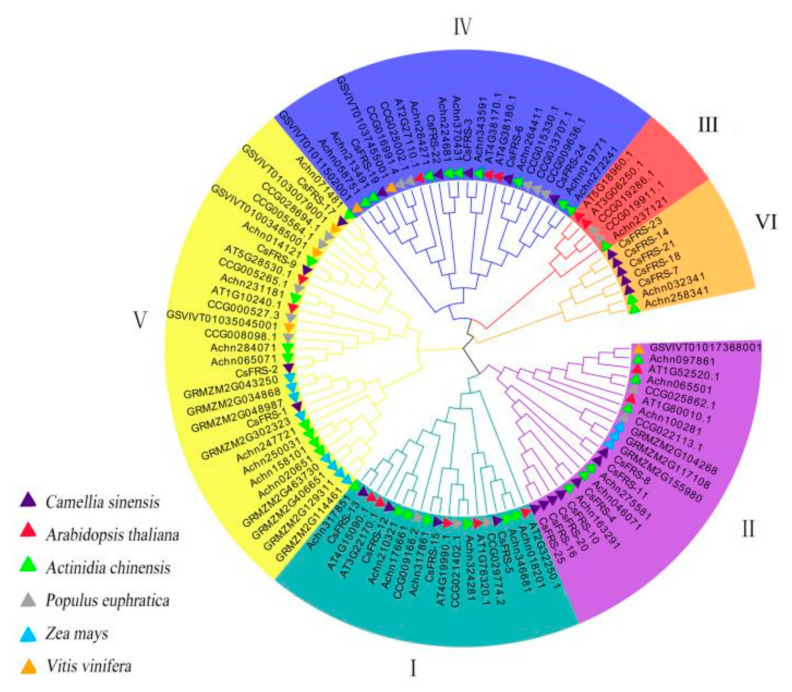
Phylogenetic analysis of the CsFHY3/FAR1 family in tea, Arabidopsis, grape, poplar, maize, and kiwifruit.

In order to further reveal the phylogenetic relationship of these gene family members, an unrooted tree of 107 FHY3/FAR1s (25 for *C. sinensis*, 14 for *Arabidopsis thaliana*, 6 for *Vitis vinifera*, 34 for *Actinidia chinensis*, 11 for *Zea mays* and 17 for *Populus euphratica*) was constructed using MEGA 7.0 software. These proteins were divided into six groups based on their sequence similarity (Figure 1), and the 25 CsFHY3/FAR1s were distributed into five groups, with four members in group I, seven members in group II, five members in group IV, four members in group V, and five members in group VI. With respect to *Z. mays* (monocotyledons), its ZmFHY3/FAR1s were distributed only in the two largest groups, groups II and V, which suggests that these two groups might be more ancient than the other groups. In contrast, the member number of group III (five for three species) and group VI (seven for two species) was lower than that of the other four groups, and there was no corresponding homologous gene in group III for *C. sinensis*, and only the protein members of the tea plants and kiwifruit were present in group VI. This indicates that groups III and VI might have evolved recently. The high number of gene duplication events of the FHY3/FAR1 family in *C. sinensis* (25 family members) and *A. chinensis* (34 family members) makes it easy to produce new functional protein isoforms, and means that the relationship between tea and kiwifruit is closer than that between tea and other species.

### 2.3. Gene Structure and Motif Analysis

**Figure 2:**
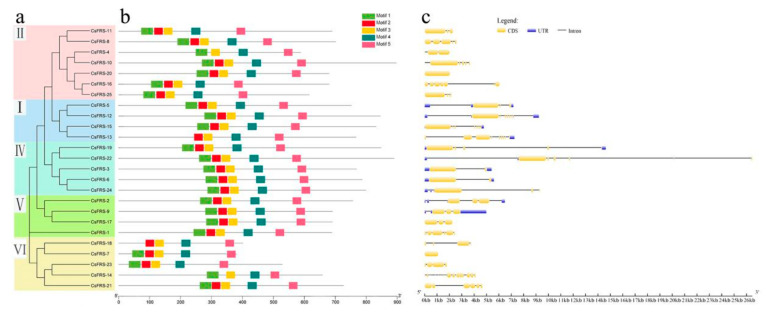
Comparative gene structure and motif analysis of CsFHY3/FAR1s. (**a**) Phylogenetic analysis and classification of CsFHY3/FAR1s. The subtree branch lines are colored in order to indicate the different subfamilies. (**b**) Motif analysis of CsFHY3/FAR1s. The top five motifs identified from the tea plant proteins obtained by the MEME analysis. (**c**) Exon-intron structures of *CsFHY3/FAR1s*. The exons are marked as yellow boxes, and the introns are represented by black lines; UTRs are shown as blue boxes.

Introns and exons are the two primary elements of genes; their numbers, length, and organization can affect gene expression levels and functions [38,39]. Therefore, it is also worthwhile to investigate the organization patterns of the 25 *CsFHY3/FAR1* family members. The analysis results obtained using GSDS2.0 are shown in Figure 2c; the intron size of groups II, V, and VI was much smaller than that of groups I and IV. The point where the promoter sequences are located, 5′ UTR, plays important roles in gene regulation, and 3′ UTR is the binding site of the mRNA degradation complex related to the stability of mRNA. In the *CsFHY3/FAR1* family, UTR sequences were observed in genes coding for groups II, IV, and V, except for *CsFRS-1* and *CsFRS-17*, while there were no UTR sequences in the genes in groups I and VI. In addition, there was great diversity in the intron numbers in the different groups, such as three to eight introns in group I, fewer than six introns in group II, and more than 20 introns for *CsFRS-14* in group VI, whereas groups IV and V had fewer than four introns. This gene structure variance indicated that introns might be acquired or constantly lost in the evolutionary process of *CsFHY3/FAR1* family members.

The conserved motif of the CsFHY3/FAR1s was further analyzed using the MEME online tool. The top five conserved motifs, motifs 1–5, are listed (Figure 2b and Table S3). All of the members except for CsFRS-4 (group II), CsFRS-13 (group I), CsFRS-18 (group VI), and CsFRS-14 (group VI) had these five conserved motifs. Motif 3, motif 4, and motif 5 were present in all of the CsFHY3/FAR1s; these motifs form the MULE and SWIM protein domains, which are the structural basis of the biological function of CsFHY3/FAR1 proteins. Noticeably, there was more variation in the conserved motif in group VI, which was specific to tea, and this may be related to the duplication and differentiation of the gene family.

### 2.4. Analysis of Cis-Acting Elements in the Promoters of CsFHY3/FAR1

**Figure 3:**
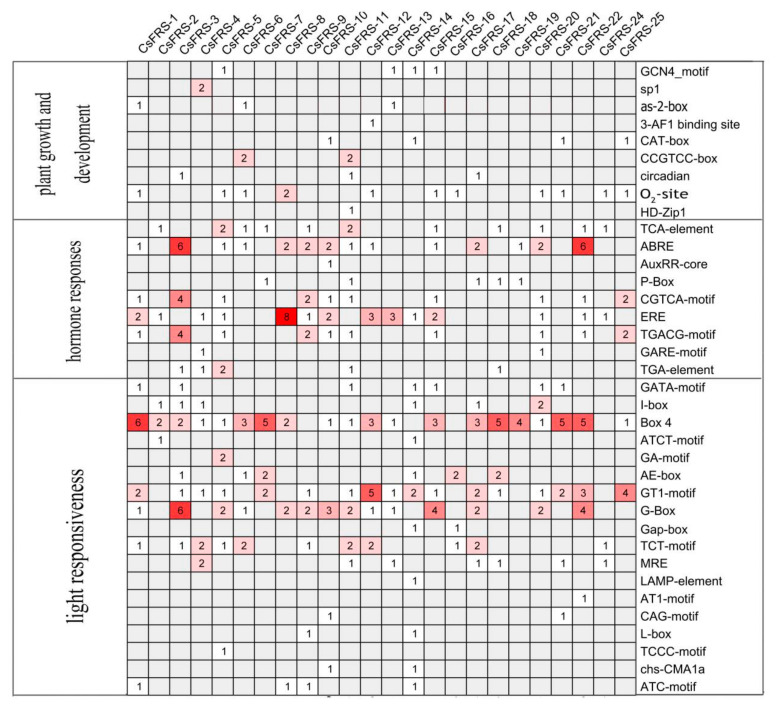
*Cis*-acting elements in promoters of *CsFHY3/FAR1s*.

*Cis*-acting elements often determine the function of genes. In order to explore the *cis*-acting elements of the *CsFYH3/FAR1* gene family, the 1.5 kb genomic sequence upstream of each gene was extracted (Table S4) and matched to the PlantCARE database [40]. The *cis*-acting elements of 24 *CsFYH3/FAR1* genes were analyzed, except for *CsFRS-23*, because its promoter region was not identified in the tea genome. The *cis*-acting elements of the *CsFYH3/FAR1s* are listed in Figure 3. Nine *cis*-acting elements were identified to be involved in plant growth and development, including GCN4_motifs, as-2-box, O_2_-site, CAT-box, and CCGTCC-box. Furthermore, a total of nine *cis*-acting elements were identified to be involved in hormonal response, such as the CGTCA motif and the TGACG motif involved in the Me-JA response; TGA-element and AuxRR-core in response to auxin; and GARE-motif and P-box in response to gibberellin. At the same time, ABRE, TCA-element, and ERE *cis*-acting elements related to abscisic acid, salicylic acid, and ethylene responses were also identified. Moreover, a total of 18 *cis*-acting elements with light-responsive components were identified, and the light-responsive *cis*-acting elements of Box 4, GT1-motif and G-Box exist in almost all of the *CsFYH3/FAR1* family members. The appearance of these *cis*-acting elements indicated that *CsFYH3/FAR1* genes may play important roles in plant growth and development, and especially in the response to light.

### 2.5. Protein Interaction Network of CsFHY3/FAR1 Proteins

**Figure 4:**
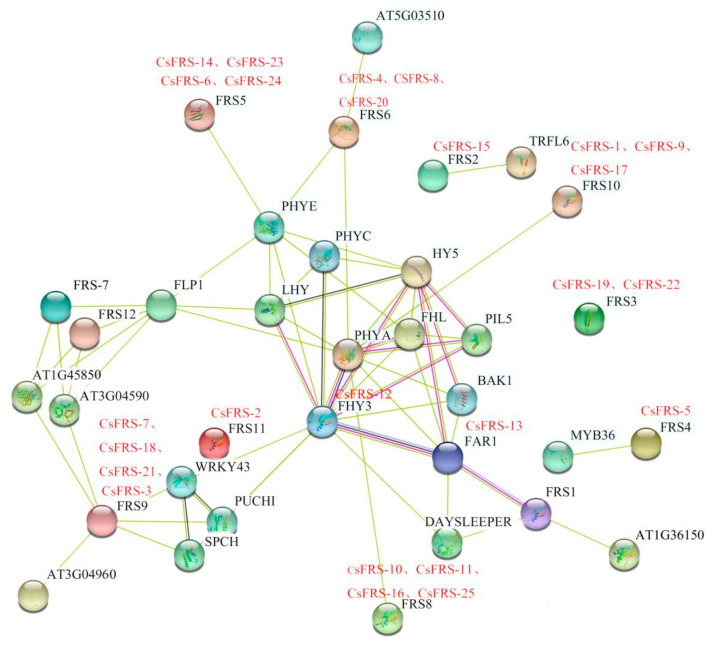
Putative interaction network of CsFHY3/FAR1s in tea plants. The homologous proteins in tea plants and Arabidopsis are shown in red and black, respectively.

The CsFHY3/FAR1s were investigated in an Arabidopsis association model using STRING software in order to identify the functional interaction. The results in Figure 4 show that 25 CsFHY3/FAR1s were mapped to 11 known Arabidopsis proteins which participate in the interaction network. Consistent with those in Arabidopsis, CsFRS-13 (corresponding to AtFAR1), CsFRS-12 (corresponding to AtFHY3), CsFRS-10, CsFRS-11, CsFRS-16, CsFRS-25, CsFRS-1, CsFRS-9, CsFRS-17, CsFRS-4, CsFRS-8, CsFRS-20, CsFRS-6, CsFRS-14, CsFRS-23, and CsFRS-24 could be integrated into the light signaling pathway through potential interaction with components of PHYA, PHYC, PHYE, and HY5. Among the CsFHY3/FAR1 family members, CsFRS-13 and CsFRS-12 are the most thoroughly studied, have the most interactions, and are closely related to PHYA and HY5. However, other members—especially CsFRS-2, CsFRS-19, and CsFRS-22—showed fewer interactions in the network. A deep investigation of these genes will expand the function and interaction network of CsFHY3/FAR1s.

### 2.6. Expression Analysis in the Different Tissues of Tea Plants

**Figure 5:**
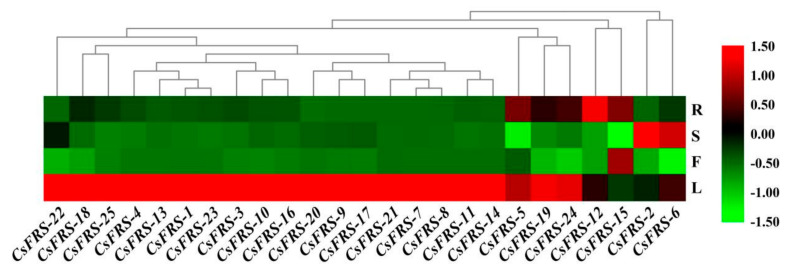
The expression profiles of *CsFHY3/FAR1s* in different tissues. R: root; S: stem; L: leaf; F: flower.

In order to investigate the tissue-specific expression pattern of *CsFHY3/FAR1s*, the expression level of 25 *CsFHY3/FAR1* genes in roots, stems, leaves and flowers was analyzed by qRT-PCR. As shown in Figure 5, the 25 *CsFHY3/FAR1s* displayed different expression patterns in different tissues of the tea plants. Most *CsFHY3/FAR1s* were highly expressed in leaves, and were poorly expressed in flowers, except *CsFRS-15*, which was highly expressed in flowers, but poorly expressed in leaves. Moreover, most *CsFHY3/FAR1s* had low expression in both shoots and roots. In contrast, *CsFRS-2* and *CsFRS-6* were highly expressed in shoots, and *CsFRS-15*, *CsFRS-12*, and *CsFRS-5* were highly expressed in roots. The expression pattern variation indicated the different regulatory roles in different plant tissues. The data are shown in Table S5.

### 2.7. Expression Analysis of CsFHY3/FAR1s under Different Stresses

**Figure 6:**
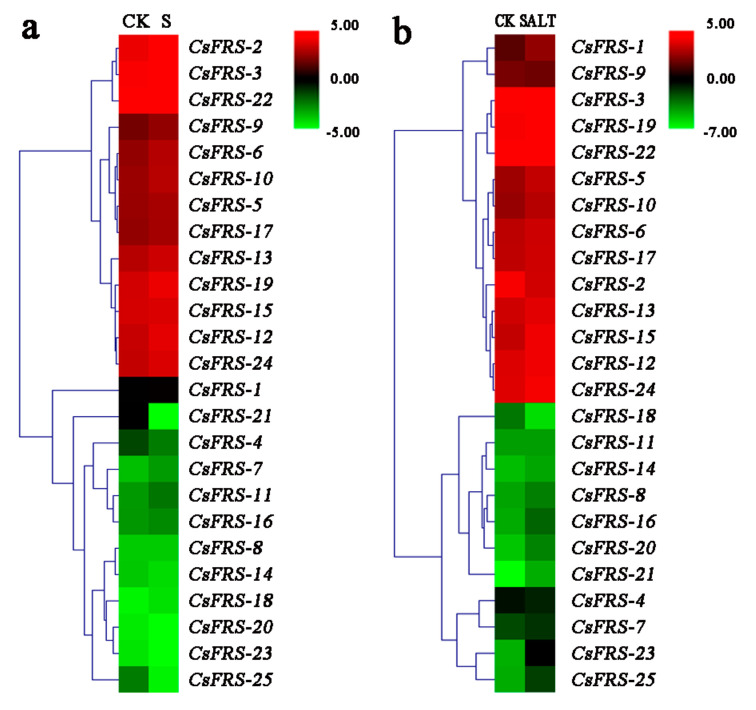
Expression of tea plant CsFHY3/FAR1 transcripts in response to salt (**a**) and shade (**b**) stress. CK: control; S: shade stress; SALT: salt stress.

The transcriptome data of *C. sinensis* under shading [41] and salt-stress treatment [42] were downloaded from the NCBI SRA (Sequence Read Archive) database. As shown in Figure 6, most of the *CsFYH3/FAR1s* had similar expression patterns under both stresses. All of group VI, most of the group II members, and *CsFRS-2* (group V) showed poor expression, compared with a higher expression level in all of the group I and group IV members. Together with the above subcellular localization prediction information, the lower expression level of *CsFRS-1* (group V), *CsFRS-23* (group II), and most of the group VI members, which was consistent with the chloroplast-targeting prediction value, indicated that the regulation events presented here might not be active. The data are shown in Table S6.

**Figure 7:**
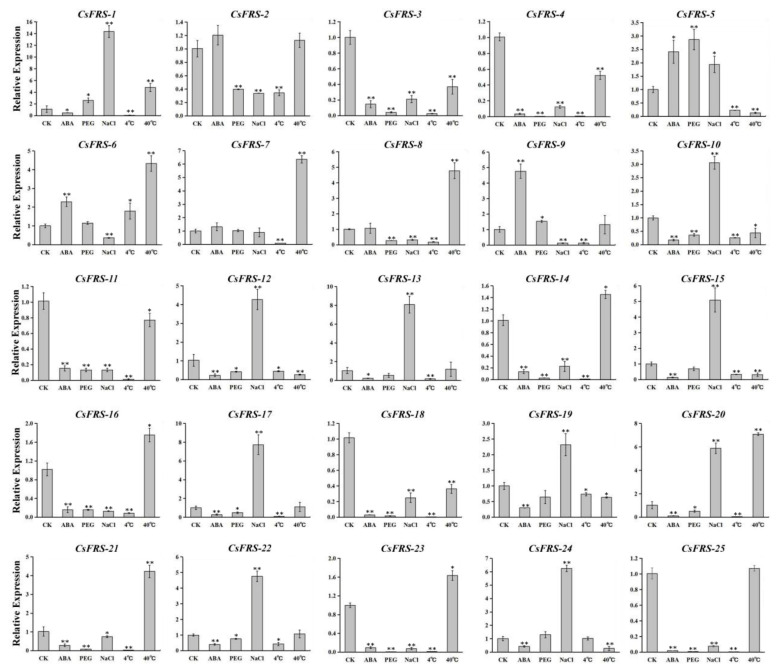
Expression analysis of the *CsFHY3/FAR1* genes in tea plants under ABA, PEG, NaCl, low temperature, and high temperature treatments. The results are expressed as the mean ± standard deviation. The asterisks (* significant, and ** highly significant) denote significant variation (*p* < 0.05).

In order to further investigate the responses of *CsFYH3/FAR1s* to biotic and abiotic stresses, the expression levels under 200 mM NaCl, high temperature (40 °C), 100 μM ABA, 20% polyethylene glycol (PEG) and low temperature (4 °C) treatments were analyzed through quantitative RT-PCR (qRT-PCR). As shown in Figure 7, 25 *CsFYH3/FAR1s* showed different responses to these stresses. The expressions of 11 *CsFYH3/FAR1s* were significantly upregulated under salt stress; these were mainly members of groups I and IV. Under high temperature stress, the expressions of 8 *CsFYH3/FAR1s* were upregulated, which were mainly members of groups II and VI. In addition, treatment with ABA, PEG, and low temperatures also resulted in the higher expression of *CsFRS-1*, *CsFRS-2*, *CsFRS-5*, *CsFRS-6*, *CsFRS-7*, *CsFRS-8*, *CsFRS-9*, and *CsFRS-24*, while the expression levels of the other *CsFYH3/FAR1s* were downregulated. The data are shown in Table S7.

The above results indicate that 25 *CsFHY3/FAR1s* with different targeting information showed specific expression responses to various external stresses. They all have light-responsive *cis*-acting elements and the main regulation motif. It is suggested that these protein family members might function differently in tea plants.

## 3. Discussion

Red and far-red light are important environmental factors which regulate plant growth and development, especially photomorphogenesis. PHYA is believed to be the main receptor which receives and responds to red and far-red light. Transposase-derived proteins FHY3/FAR1 modulate the PHYA entry into the nucleus by directly activating the expression of *FHY1* and *FHL*. In addition, AtFHY3, AtFAR1, and 12 other AtFRSs have been identified in Arabidopsis, and have high homology in terms of structure, morphology, and functions [15]; these are indispensable elements for the maintenance of the normal growth of plants. However, the homologous genes have not been identified or characterized in tea plants.

In this study, 25 *CsFHY3/FAR1* coding sequences were identified in the genome of *C. sinensis* var. *sinensis* [37] (Table 1 and Table S2), and then their physicochemical properties and subcellular localization were analyzed and predicted. Transcription factors mainly function in the nucleus to regulate gene expression. However, some of the identified CsFHY3/FAR1 family members were predicted to target other cell components, such as CsFRS1, CsFRS7, CsFRS14, CsFRS16, CsFRS18, and CsFRS23 (Table 1). In Arabidopsis, AtFRS1, AtFRS8, and AtFRS9 are also predicted to lack a putative nuclear localization sequence, but they can still target the nucleus, and have a non-typical nuclear localization sequence or interact with other members; the nuclear localization sequence of FRS proteins can be co-imported into the nucleus [15]. This suggested that although some of the CsFHY3/FAR1s were not be predicted to occur in the nucleus, they might enter the nucleus by interacting with other members of CsFHY3/FAR1s to form a complex. The specific subcellular localization of CsFHY3/FAR1 needs further experimental verification.

Based on multiple sequence alignment and phylogenetic analysis, FHY3/FAR1 family proteins from six different species were divided into six groups (Figure 1 and Figure 2). The 25 CsFHY3/FAR1s were mapped to five groups; in group III, there was no corresponding locus for *C. sinensis*. In addition, group VI was closely related to groups III and IV, but was older than these two groups. This suggests that group VI might be unique to tea plants and kiwifruit. Gene duplication events are very common in the process of plant evolution. The divergence of the tea and kiwifruit lineages occurred 80 million years ago, and tea plants underwent two duplication events compared with the diploid grape genome [37]. Duplication events were also observed in the FHY3/FAR1 family, such as FRS5, FRS6, FRS8, FRS9, and FRS10 in Arabidopsis and tea plants (Figure 1, Figure 2 and Figure 4). The deletion of FRS1, FRS7, and FRS12 in tea plants was also found (Figure 1 and Figure 4). These phylogenetic tree and cluster analysis results indicated that duplication and deletion events occurred in the CsFHY3/FAR1 family in the evolutionary process.

The conserved protein motifs and gene structures of the CsFHY3/FAR1 family were further characterized. The structural characteristics of genes in the evolutionary process of plants have always been an important molecular basis for plants to adapt to environmental changes, and are important manifestations of different groups of gene families [43]. In the intron/exon structure of *CsFHY3/FAR1s*, each group has similar structural features: group I has a longer intron length, with a number between five and eight; group II genes have two to five shorter introns, and the exon distribution is concentrated; in groups IV, V and VI, except for *CsFRS-14*, the number of introns is within five, and groups IV and V have a UTR area, whereas group IV has a longer intron length (Figure 2). In general, the intron density is mostly at a low level, which contributes to stress regulation [44]. The motifs are also very similar; motifs 3, 4 and 5 are present in each gene, and are important components of MULE and SWIM, as is consistent with findings in Arabidopsis [15].

Previous studies on Arabidopsis the FHY3/FAR1 family proteins have been limited to FAR1 and FHY3. AtFHY3 and AtFAR1 participate in plant growth and development, by directly or indirectly interacting with *PHYA*, *FHL*, *HY5*, *CCA1*, *SPLs*, *ARC5*, *HEMB1*, *ISA2*, *PIF1*, and/or *EIN3* [9,11,13,16,22,27]. The analysis of *cis*-acting elements and the prediction of the protein interaction network of 25 CsFHY3/FAR1 family members indicated the enrichment of *cis*-acting elements, such as as-2-box, O_2_-site, ABRE, ERE, HD-Zip1, Box 4, GT 1-motif, or G-Box, and the interaction network together with PHYA, PHYC, PHYE, or HY5 suggested that CsFHY3/FAR1 family members also play wide roles in the light response, hormone response, and growth and development of tea plants.

It has been reported that *AtFHY3/FAR1* family genes exhibit different expression patterns in their rosette leaves, cauline leaves, stems, flowers, and siliques, while *AtFRS10* was detected in the hypocotyl and cotyledons using the FRS10:GUS reporter gene [15]. In addition, in the tissue-specific expression analysis of cotton, most Ga*FHY3/FAR1* family genes were highly expressed in leaves, but were poorly expressed in other tissues, such as stems and the torus [45]. In this paper, the tissue-specific expression analysis results of the *CsFHY3/FAR1* family showed that almost all of the members were highly expressed in leaves, which is an important tissue that receives and responds to light signals, suggesting that these *CsFHY3/FAR1s* might be responsible for this process. These results are consistent with those obtained for Arabidopsis and cotton [11,45]. Moreover, *CsFRS-2* and *CsFRS-6* were highly expressed in the stem, *CsFRS-15* was highly expressed in the flower, and *CsFRS-12* was highly expressed in the root. These tissue-specific expression patterns revealed that *CsFHY3/FAR1s* might function differently in different tissues (Figure 5).

FHY3 and FAR1 bind directly to the promoter of *ABI5*, and are involved in ABA signaling in Arabidopsis [11,28]. In our study, under ABA treatment, three genes were upregulated and 19 genes were down-regulated in the CsFHY3/FAR1 family. The down-regulation of 19 CsFHY3/FAR1s might prevent the damage caused by a high concentration of ABA, and might reduce stress, which is consistent with a previous study [28]. Abiotic stresses such as drought, salt and high temperatures can produce a high number of reactive oxygen species in plants. Recent reports indicate that AtFHY3 and AtFAR1 negatively regulate the accumulation of reactive oxygen species [29,30]. In our study, 17 genes were down-regulated in the PEG treatment, which indicates that *CsFHY3/FAR1s* may also be involved in the control of the accumulation of reactive oxygen species. In addition, almost all of the genes (23/25) were inhibited at a low temperature, which—similar to CsbZIP18—might be due to the low transcription activity of the transcription complex and/or some of these *CsFHY3/FAR1s* participating in low-temperature responses [46].

## 4. Conclusions

In this study, we comprehensively and systematically analyzed the FHY3/FAR1 family in *C. sinensis*. In total, 25 *CsFHY3/FAR1* genes were identified, their phylogenetic and gene structures were analyzed, and the *cis*-acting elements and protein interaction network were predicted. The expression of 25 *CsFHY3/FAR1s* in different tissues or under different stresses was determined. These results indicate that *CsFHY3/FAR1s* might be involved in regulating photomorphogenesis, growth and development, and abiotic stresses by regulating downstream responses. These results will provide the foundation for additional functional studies investigating the CsFHY3/FAR1 family, and will contribute to an improved understanding of the mechanisms of light and stress tolerance mediated by CsFHY3/FAR1 in tea plants.

## 5. Materials and Methods

### 5.1. Plant Materials and Stress Treatments

One-year-old cut seedlings of tea plants (*C. sinensis* cv. ‘*Fudingdabai*’) were grown in a chamber at Northwest A&F University (Yangling, China) under a 12 h photoperiod at 25 °C during the day and 20 °C at night. Cut seedlings with strong and uniform growth were selected for the different biotic and abiotic stress treatments for 8 h, including NaCl, ABA, drought, heat and cold stresses [47]. For the heat and cold treatments, the tea plants grown under normal conditions were transferred to an artificial climate chamber maintained at 40 °C or 4 °C. For the salt and drought treatments, the roots of the tea plants, together with the medium, were immersed completely in a solution containing 200 mM NaCl and 20% (*w*/*v*) polyethylene glycol (PEG) 6000. For the ABA treatment, 100 uM ABA was sprayed onto the tea leaves. The first and second tender leaves of the treated tea plants were collected. For the tissue-specific expression analysis, roots, stems, leaves and flowers were collected from the cut seedlings. All of the treatments were completed under consistent growth conditions, and each treatment had three biological replicates. All of the samples were rapidly frozen in liquid nitrogen and stored at −80 °C for further analysis.

### 5.2. Identification of the CsFHY3/FAR1 Gene Family in Tea Plants

In order to identify the *CsFHY3/FAR1* family genes in tea plants, PF03101, PF10551 and PF04434, which are labeled *FHY3/FAR1* gene family domains, were downloaded from the Pfam database (http://pfam.xfam.org/), and were used as queries in the HMMER Version 3.3 (http://www.hmmer.org/); the tea plant genome was downloaded from the Tea Plant Information Archive (TPIA, http://tpia.teaplant.org) [37]. Then, the obtained *CsFHY3/FAR1* genes were rechecked using the NCBI CDD (Conserved Domains) database (https://www.ncbi.nlm.nih.gov/cdd) and Interpro (https://www.ebi.ac.uk/interpro/) in order to remove non-family members and repetitive sequences.

### 5.3. Sequence Analysis and Phylogenetic Tree Construction

The physiological and biochemical properties—including the number of amino acids, molecular weight (MW), theoretical isoelectric point (pI), aliphatic index, tgrand average of hydropathicity (GRAVY), and instability index—of the CsFHY3/FAR1s were analyzed using ExPASy ProtParam (http://www.expasy.org/tools/protparam.html). The WoLF PSORT program (https://wolfpsort.hgc.jp/) was used to predict the subcellular localization of the CsFHY3/FAR1s. The protein sequences of AtFHY3/FAR1s were downloaded from the TAIR website (https://www.arabidopsis.org/). The FHY3/FAR1 family protein sequences of grape, poplar, maize and kiwifruit were downloaded from PlantTFDB v5.0 (planttfdb.gao-lab.org/index.php); the non-family members and repetitive sequences were removed, and a phylogenetic tree was constructed with the FAR1/FHY3 family proteins of tea and Arabidopsis. The phylogenetic tree was constructed using the neighbour-joining (NJ) method with 1000 bootstrap replicates in MEGA 7.0 [48].

### 5.4. Analysis of Gene Structure and Conserved Motifs

GSDS (Gene Structure Dispaly Server) [49] (http://gsds.cbi.pku.edu.cn/) was used to analyze the exon-intron structures of the *CsFAR1/FHY3s*. The conserved motifs of the CsFHY3/FAR1s were analyzed by MEME Version 5.0.5 (meme-suite.org/tools/meme), with the maximum number of motifs set to five, and with the other parameters as the default.

### 5.5. Prediction of the Cis-Acting Elements and Protein Interaction Network

The sequence 1500 bp upstream of the *CsFHY3/FAR1s* was extracted from the tea plant genome [37,50]. The *cis*-elements of the promoter regions were screened by PlantCARE (http://bioinformatics.psb.ugent.be/webtools/plantcare/html/). The functional interaction network models of the CsFHY3/FAR1 family proteins were predicted using STRING (https://string-db.org/), with the confidence parameter set to a threshold of 0.40.

### 5.6. Transcriptome Analysis

The transcriptomes of leaves from the shading (BioProject: PRJNA356134) [41] and the salt-stressed (BioProject: PRJNA387271) [42] treatments were downloaded from the NCBI SRA database (http://www.ncbi.nlm.nih.gov/sra/), and were used to analyze the expression levels of *CsFHY3/FAR1s*. The expression levels of *CsFHY3/FAR1s* were calculated using the fragment per kilobase million (FPKM) method. In order to visualize the expression data, a heatmap of the gene expression was created using MultiExperiment Viewer (MeV).

### 5.7. Quantitative Real-Time PCR (qRT-PCR) Analysis

A biospin polysaccharide polyphenol extraction kit (Bioflux, Beijing, China) was used to extract the total RNA from the first and second tender leaves of tea plants after the biotic and abiotic stress treatments, and different tissues for tissue-specific expression analysis, then the concentration of the RNA samples was measured using a NanoDrop ND 1000 spectrophotometer (Thermo Fisher Scientific, Waltham, MA, USA). The integrity of the RNA samples was observed by agarose gel electrophoresis using 1 μg total RNA to synthesize the first-strand cDNA according to a 5× All-In-One RT MasterMix Kit (ABM, Richmond, Canada). Subsequently, the cDNA samples were diluted to 50 ng/μL using RNase-free ddH_2_O. ChamQ SYBR qPCR Master Mix (Vazyme, Nanjing, China) was used for the qRT-PCR on an IQ5 Real-Time PCR System (Bio-Rad, Hercules, USA). The *Csβ-actin* gene (GeneBank: KJ946252) was used as a reference gene [51]. All of the primers used for the qRT-PCR analysis were designed by Primer-Blast (https://www.ncbi.nlm.nih.gov/tools/primer-blast/), and are listed in Table S1. Three independent biological replicates and technical replicates for each sample were analyzed. The relative expression levels were calculated according to the 2^−∆∆Ct^ method [52]. The heatmap of the gene expression was created using MeV.

### 5.8. Statistical Analysis

The data are presented as the mean values and standard deviations (SD) of three biological and technical replicates. A *t* test was used to determine the significant differences among the given treatments. A *p* value of < 0.05 was considered statistically significant. All of the statistical analyses were performed using Excel (Microsoft Corp., Redmond, WA, USA) and Sigmaplot 12.5 (Softonic International, Barcelona, Spain).

## Figures and Tables

**Table 1:** Summary information of the physiological and biochemical properties of the CsFHY3/FAR1.

Gene Name	Gene ID	Amino Acids	MW (kDa)	pI	GRAVY	Instability Index	Aliphatic Index	Subcellular Location
*CsFRS-1*	CSS002622.1	689	80.13	9.10	−0.434	46.00	77.21	chlo:4, vacu:3, cyto:2, nucl:1.5, cysk_nucl:1.5, mito:1, plas:1, extr:1
*CsFRS-2*	CSS016876.1	757	86.03	6.20	−0.318	47.61	77.64	nucl:13, vacu:1
*CsFRS-3*	CSS003256.1	769	87.81	5.79	−0.509	53.55	72.65	nucl:13, cyto:1
*CsFRS-4*	CSS001271.1	588	68.71	8.91	−0.560	48.43	68.59	nucl:7, pero:2, cysk:1.5, cysk_plas:1.5, cyto:1, mito:1, vacu:1
*CsFRS-5*	CSS001125.1	752	86.58	6.47	−0.539	50.51	76.77	nucl:13, vacu:1
*CsFRS-6*	CSS013735.1	787	90.41	6.26	−0.565	52.42	71.60	nucl:14
*CsFRS-7*	CSS042110.1	368	43.19	8.42	−0.428	39.24	65.76	cyto:7, cysk:5, nucl:1, golg:1
*CsFRS-8*	CSS022313.1	702	80.33	9.28	−0.385	41.50	80.24	nucl:11, cyto:2, cysk:1
*CsFRS-9*	CSS018104.1	691	80.13	6.04	−0.351	42.06	77.54	nucl:7, chlo:4, mito:2, cysk:1
*CsFRS-10*	CSS039560.1	897	103.05	7.36	−0.568	49.04	70.51	nucl:13, plas:1
*CsFRS-11*	CSS007312.1	690	79.47	9.06	−0.436	39.05	70.04	nucl:12, cyto:1, cysk:1
*CsFRS-12*	CSS040860.1	846	97.07	6.98	−0.687	52.72	63.43	nucl:14
*CsFRS-13*	CSS040864.1	767	88.40	7.38	−0.692	57.85	66.75	nucl:6, pero:4, chlo:2, cyto:1, mito_plas:1
*CsFRS-14*	CSS035662.1	659	75.35	9.11	−0.428	39.71	68.21	chlo:9, nucl:4, vacu:1
*CsFRS-15*	CSS052639.1	832	95.68	7.49	−0.501	54.13	74.13	nucl:13, vacu:1
*CsFRS-16*	CSS022572.1	680	79.60	8.97	−0.558	47.81	77.25	cyto:8, nucl:4, mito:1, plas:1
*CsFRS-17*	CSS003022.1	691	79.70	6.03	−0.357	44.10	79.58	nucl:6, mito:3.5, cyto_mito:2.5, chlo:2, cysk:2
*CsFRS-18*	CSS008927.1	402	47.13	8.91	−0.491	48.24	67.69	cyto:8, nucl:2, plas:2, mito:1, pero:1
*CsFRS-19*	CSS040724.1	848	96.73	8.70	−0.525	45.91	70.06	nucl:13, cyto:1
*CsFRS-20*	CSS025022.1	680	79.13	6.56	−0.594	55.50	68.50	nucl:13, vacu:1
*CsFRS-21*	CSS052868.1	727	83.96	8.33	−0.583	35.12	64.68	nucl:12, plas:1, vacu:1
*CsFRS-22*	CSS032048.1	890	101.02	6.70	−0.532	44.93	67.07	nucl:12, cyto:2
*CsFRS-23*	CSS025195.1	529	61.07	8.92	−0.460	41.99	65.80	chlo:11, nucl:2, mito:1
*CsFRS-24*	CSS043153.1	799	90.80	6.04	−0.467	47.21	72.60	nucl:13, chlo:1
*CsFRS-25*	CSS050875.1	616	71.73	6.65	−0.501	47.28	76.25	nucl:9, cyto:4, plas:1

chlo: chloroplast; cysk: cytoskeleton; cyto: cytoplasm; extr: extracellular; golg: Golgi apparatus; mito: mitochondrion; nucl: nucleus; pero: peroxide; plas: plasma membrane; vacu: vacuole.

## Data Availability

The data presented in this study are available in the article and its supplementary materials.

## Supplementary Materials

The following are available online at https://www.mdpi.com/2223-7747/10/3/570/s1. Table S1: Primers used for the qRT-PCR of *CsFHY3/FAR1* genes. Table S2: The coding DNA sequences (CDSs) and deduced amino acid sequences of the *CsFHY3/FAR1* genes. Table S3: The motif sequences of the CsFHY3/FAR1 family proteins. Table S4: The sequence of 1500 bp upstream of the *CsFHY3/FAR1s* genes. Table S5: Expression levels of the *CsFHY3/FAR1* genes in four different tissues from tea plants. Table S6: Analysis of the tea plant *CsFHY3/FAR1* transcription levels in response to salt and shade stress. Table S7: Expression levels of the *CsFHY3/FAR1s* in tea plants following the different treatments.
